# Concours for the Chair of Clinical Surgery, in the Academy of Medicine, Vacant by the Death of Prof. Augustus Bérard

**Published:** 1848-02

**Authors:** 


*Concours for the Chair of Clinical Surgery, in the Academy of Medicine, vacant by the death of Professor Aug. Bérard.*—The concours was opened on the 15th of November. The following gentlemen are candidates for the chair:—MM. Alequié, P. Boyer, Chassaignac, Laugier, Maisonneuve, Malgaigne, Michon, Robert, Vidal, and Sanson.

The first part of the competition consisted in a written question, for the composition of which five hours were granted. The subject was "The History of Strictures." Each paper was read in the amphitheatre, before the jury and a crowded audience. The first clinical "épreuve" was then instituted; it consists in a lecture of one hour, delivered in the amphitheatre of the faculty, immediately after examination of two patients chosen in the surgical wards of La Charité or the Hôtel Dieu, a quarter of an hour being allowed for the examination of each patient. But the public, not being admitted into the hospital during this first stage of the proceedings, remains ignorant of the correct diagnosis of the cases, and can only form an opinion of the general merits of the lectures. It also happens, on account of the short time granted for the explication of the two cases, that the candidate does not always find sufficient matter for a lecture of one hour's duration, and, to fill up the time, is compelled to make a more or less interesting disquisition on the malady, instead of a special history of the individual case. We have, further, heard very excellent lectures delivered upon an incorrect diagnosis, and reciprocally a correct and difficult diagnosis accompanying a lecture of very ordinary merits. Several candidates have already gone through this first clinical "épreuve," and, from the mere consideration of the concours itself, it is hitherto perfectly impossible to form any idea of its probable issue, although the initiated speak strongly of the predisposition of the jury in favour of one candidate.—*Med. Times.*

